# Hidden Triggers of Degradation during Fabrication of Inorganic Perovskite Solar Cells

**DOI:** 10.1021/acsami.5c22948

**Published:** 2026-02-19

**Authors:** Vladimir Shilovskikh, Herman Heffner, Yitian Du, Zongbao Zhang, Fabian Paulus, Boris Rivkin, Yana Vaynzof

**Affiliations:** † Chair for Emerging Electronic Technologies, TUD Dresden University of Technology, Nöthnitzer Str. 61, 01187 Dresden, Germany; ‡ Leibniz Institute for Solid State and Materials Research Dresden, Helmholtzstraße 20, 01069 Dresden, Germany

**Keywords:** perovskite solar cells, indium tin oxide, degradation, laser patterning, chemical etching, CsPbI_3_

## Abstract

Photovoltaic devices based on inorganic perovskites, such as CsPbI_3_, are of great interest for applications, either as a single-junction or in Si/perovskite tandem devices due to their favorable bandgap. Such applications often require the deposition of the perovskite active layer on patterned indium tin oxide (ITO) layers. Yet, in many instances, the deposition of CsPbI_3_ on structured ITO leads to the almost instantaneous degradation of the perovskite layer during film formation. In this work, we demonstrate how the microstructural and topographical features of patterned ITO substrates influence the degradation of CsPbI_3_ into its nonperovskite δ-phase. By comparing two methods for patterning ITO, i.e., laser-patterning and chemical etching, we demonstrate that perovskite degradation consistently initiates at laser-formed terminations. We utilize scanning electron microscopy, electron backscatter diffraction, and confocal microscopy to prove that even nanoscale surface steps and microcrater edges, approximately 50 nm in height, are sufficient to trigger localized δ-phase formation. These regions exhibit distinct thermal and structural behavior, including recrystallization and grain coarsening. Our study provides a mechanistic understanding of how substrate morphology drives phase instability during film growth, paving the way for substrate engineering strategies to suppress phase instabilities that occur during the fabrication of inorganic perovskite-based optoelectronic devices.

## Graphical abstract

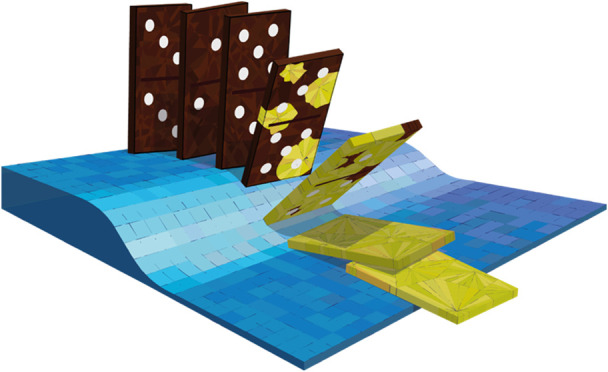

## Introduction

Perovskite solar cells (PSCs) are among the most promising emerging photovoltaic technologies due to their remarkably high efficiency, low production costs, and versatile applications.^1,2^ Among the various perovskite compositions, all-inorganic perovskites such as cesium lead triiodide (CsPbI_3_) have gained significant interest due to their promising thermal stability and potential for highly performing devices.^3^ The power conversion efficiency (PCE) of CsPbI_3_-based PSCs has achieved a significant milestone at 22% through recent research advances.^4^

Research on perovskite solar cells extensively utilizes substrates with patterned transparent conductive oxide strips, such as indium tin oxide (ITO), which combine conductive and insulating areas to define active device regions.^5^ The insulating areas are typically created by removing ITO from the underlying substrate through chemical etching or laser ablation processes. Chemical etching is achieved by exposing the desired regions of ITO to highly acidic solutions,^6^ while laser ablation uses high-energy laser pulses to create precise patterns.^7^ Chemical etching of ITO has been extensively optimized over the last three decades and is a well-established procedure with minimal edge artifacts, such as defects or undercuts.^8–10^ On the other hand, it suffers from some disadvantages, as chemical etching rates may vary drastically based on the crystalline structure of ITO,^11^ and the etchants (hydrochloric acid or ferric chloride) are highly corrosive. Laser ablation is a dry process that avoids hazardous chemicals, allows for flexible pattern adjustment, and facilitates additional interface modifications.^12,13^ However, it can also lead to undesired side effects, including local heating, structural damage, and surface defects.^14^

CsPbI_3_ PSCs based on patterned ITO substrates in both the standard and inverted architectures employ interfacial layers such as NiO_*x*_,^15,16^ SnO_2_,^17–20^ ZnO,^21^ conductive polymers like P3CT-N,^22–26^ PTAA, or PEDOT/PSS,^27–31^ or organic complex interlayers to mitigate interface-related degradation.^32–35^ The thickness of such interfacial layers is typically on the order of tens of nanometers, which smoothens the structured nature of patterned ITO. However, these materials often exhibit a mismatch in energy levels with the perovskite material or an imperfect charge extraction process that needs to be addressed through surface passivation or interface engineering.^36–41^ A promising approach to solve these challenges is utilizing self-assembled molecular (SAM) transport layers as highly efficient interlayers for perovskite photovoltaics.^42,43^ Indeed, self-assembled molecular layers such as MeO-2PACz ([2-(3,6-dimethoxy-9*H*-carbazol-9-yl)­ethyl]­phosphonic acid) became increasingly popular for PSC fabrication.^44,45^ Even though these layers are known to form multilayers, they are typically significantly thinner, reproducing the texture and structure of the underlying ITO.^46^ Interestingly, the use of SAM interlayers is surprisingly rare in the case of CsPbI_3_ solar cells. Some studies have used SAMs in combination with NiO_*x*_.^47,48^ Nevertheless, to the best of our knowledge, no other group apart from ours has successfully fabricated CsPbI_3_ solar cells using only SAM as extraction layers.^49,50^ This scarcity raises questions about the inherent compatibility of CsPbI_3_ with SAMs or with the use of patterned ITO.

Unlike previous reports that focus on improving device efficiency or environmental stability, this work concentrates on the fundamental mechanisms that govern the intrinsic instability of CsPbI_3_ during film formation. The objective is to identify how substrate morphology and microstructure trigger phase transformation, providing mechanistic insight rather than a device-optimization route. In this work, we explore the formation of CsPbI_3_ films on MeO-2PACz-treated ITO substrates that were patterned using either chemical treatment (CT) or laser treatment (LT). We identify that the morphological properties of the laser-patterned ITO edges play a crucial role in triggering the degradation of CsPbI_3_ to the δ-phase already during the fabrication process. On the other hand, CT-patterned ITO does not lead to such degradation, enabling the fabrication of CsPbI_3_ devices. These findings offer critical insights into the often-overlooked challenges associated with substrate patterning in PSC fabrication, guiding future research toward more stable and reliable CsPbI_3_-based devices.

## Results and Discussion

To investigate the formation of CsPbI_3_ thin films on MeO-2PACz-treated ITO substrates, we followed an established procedure for depositing β-CsPbI_3_ using dimethylammonium iodide (DMAI)^49,50^ on either CT- or LT-ITO substrates. Both substrate types contain a patterned ITO stripe of 7 mm width, centered on a 12 mm wide substrate (see Figure S1 for a schematic structure and photographs of both substrates). Figure 1a,b displays photographs of CsPbI_3_ films deposited and annealed for different periods (1, 2, 5, and 10 min) to convert the perovskite precursors into β-CsPbI_3._ Films deposited on laser-treated ITO substrates (Figure 1a) displayed a discoloration at the edges of the ITO stripe already after 2 min of annealing, regardless of the surrounding atmosphere (Figure S2a) or the delay between film casting and annealing (Figure S2b), which progressively grew until the film was primarily transparent after 10 min. This discoloration results from a conversion to the δ-phase, as is confirmed by X-ray diffraction measurements (Figure S3). On the other hand, no such effect was observed on CT-ITO substrates (Figure 1b), where the annealing led to the formation of a progressively darker film. Dark areas on both substrates demonstrate the formation of polycrystalline perovskite areas (Figure S4). Interestingly, the behavior is specific to CsPbI_3_ and is not observed for other perovskite compositions. For example, Figure 1c,d displays the photographs of triple-cation (formamidinium/methylammonium/cesium, 0.83:0.12:0.05) perovskite films deposited on MeO-2PACz-treated LT- and CT-ITO, neither of which shows the degradation effect observed for CsPbI_3_ on LT-ITO. The consequence of this degradation is evident in comparing the performance of solar cells fabricated using the deposited layers. While CsPbI_3_ devices fabricated using CT-ITO are functional and show reasonable performance, no working devices could be made on LT-ITO. This is not the case for triple-cation devices, where both substrates yield comparable efficiencies. (Figure 1e), suggesting the effect is present only for inorganic perovskites, which are known to be more sensitive to phase instabilities.

**Figure 1:**
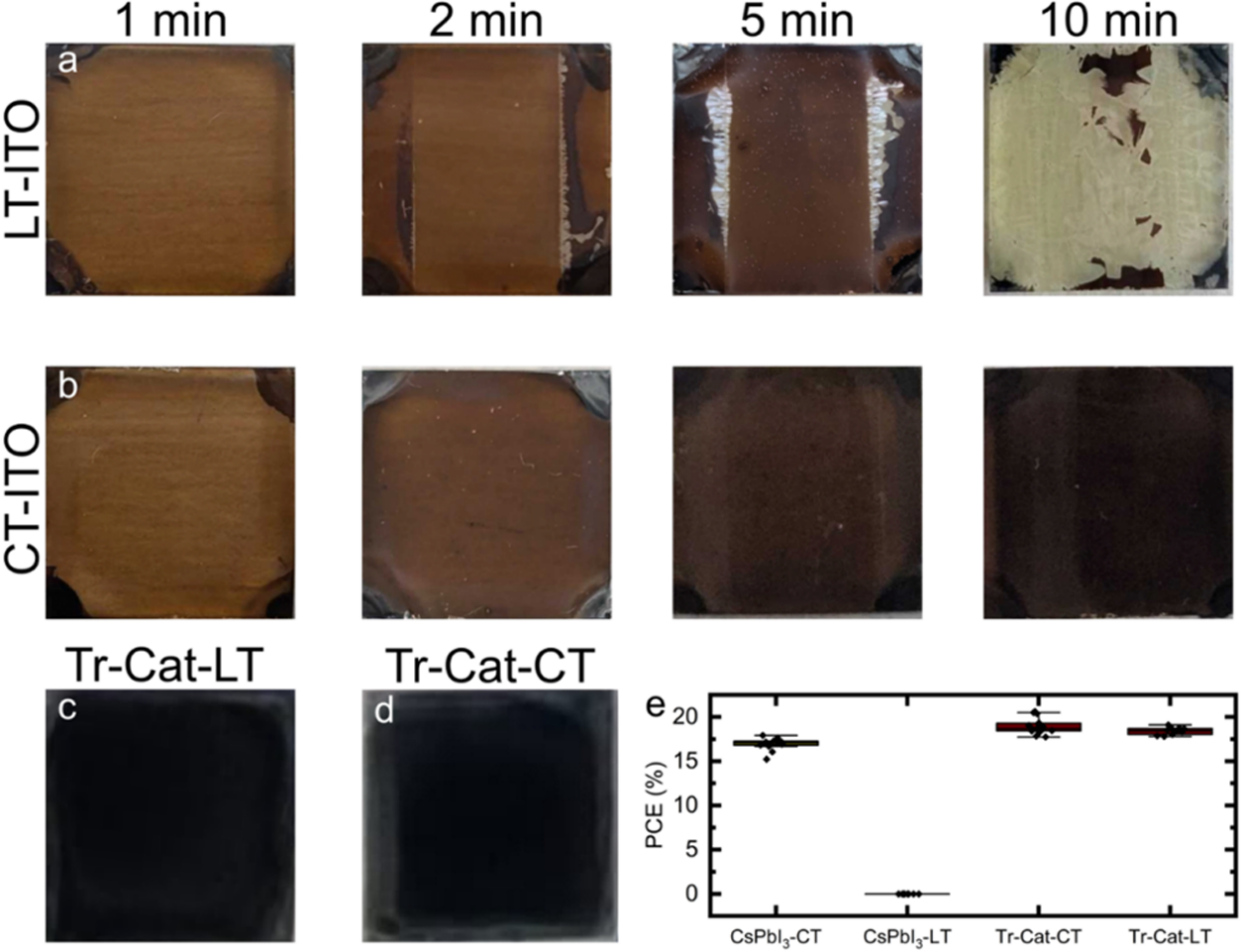
Photographs of CsPbI_3_ films after solution deposition and annealing for 1, 2, 5, and 10 min on (a) LT-ITO and (b) CT-ITO. Photographs of triple-cation perovskite films on (c) LT-ITO and (d) CT-ITO. (e) Power conversion efficiency of solar cells on CT and LT ITO substrates. Both ITO substrates have a 12 × 12 mm^2^ dimension.

To trace the degradation process that occurs at the edges of patterned LT-ITO substrates, samples at the early stages of degradation (i.e., short annealing duration) were characterized using various microscopic methods (Figure 2a–f). The transition from submicrometer tetragonal grains to orthorhombic δ-CsPbI_3_ grains, characterized by larger grains and birefringence, can be verified through polarized optical microscopy (Figure 2a).^51^ Additionally, scanning electron microscopy (SEM) can easily distinguish differences in grain size, shape, and apparent texture, allowing for visual discrimination on a microscale of the photoactive β-CsPbI_3_ from inactive δ-CsPbI_3_, and to describe the process in general (Figure 2b,c). However, to ensure an unambiguous correlation between the macroscopic appearance visible in polarized light and secondary electrons with a specific phase of CsPbI_3_, electron backscatter diffraction (EBSD) was employed as a local, direct microstructure determination method.^52^ EBSD can distinguish between the phases of the perovskite grains and estimate boundaries between two phases with a ∼10 nm resolution. It is based on electron diffraction by crystallographic planes within several nanometers of the sample’s surface and thus may provide information on the sample’s crystallinity by measuring diffraction contrast (DC) (Figure 2d). The spatial distribution of the electron diffraction maxima is unique for a given crystallographic phase (Figure 2e), and the crystallite orientation, which is coded in Euler angles due to the need to transfer a three-dimensional array to a two-dimensional plane (Figure 2f). The results of all three methods are consistent and indisputably indicate the formation of δ-CsPbI_3_ at the edges of the patterned LT-ITO.

**Figure 2:**
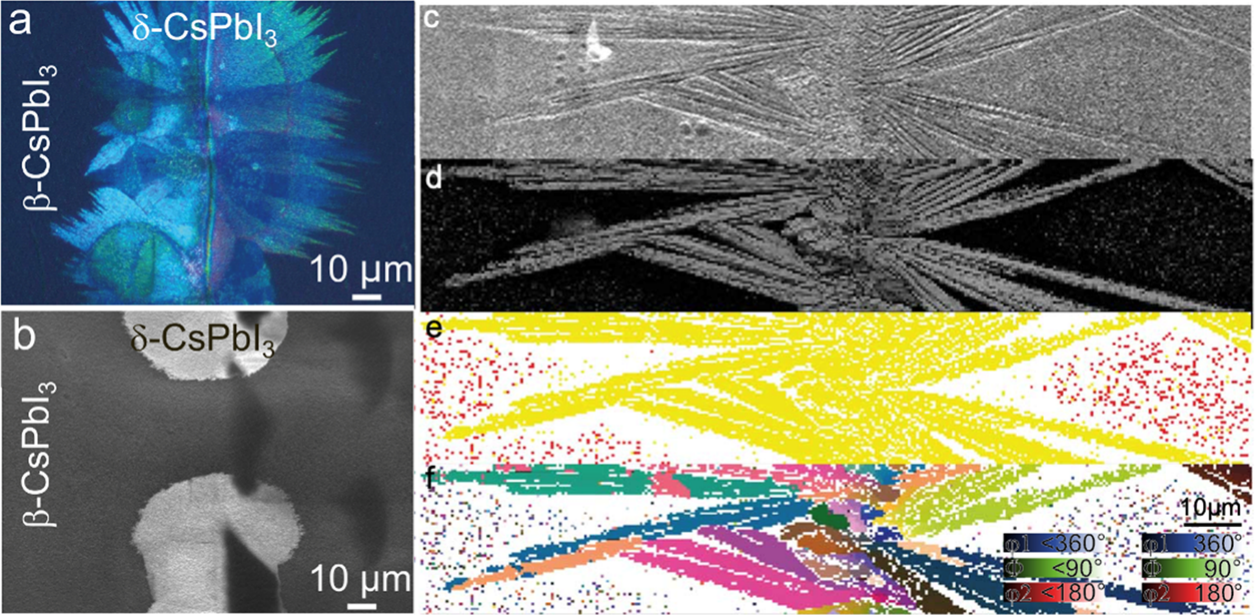
CsPbI_3_ degradation hotspots over ITO termination (termination line is vertical in the middle, ITO on the left, glass on the right) in (a) polarized light, (b) secondary electron image SEM, and (c–f) local electron microdiffraction maps (EBSD) for: (c) SEM, (d) diffraction contrast (DC), (e) phase, and (f) Euler angles.

All characterization methods consistently showed radial degradation originating from a single center defined herein as a *degradation hotspot*. The reproducible hotspot emergence of δ-CsPbI_3_ along LT-ITO boundaries, contrasting with stable film regions elsewhere, indicates that the termination of the ITO layer pattern plays a critical role in triggering this degradation and must be closely examined. SEM analysis, displayed in Figure 3a,b, shows that LT-ITO substrates exhibit features consistent with laser-induced melting, while CT-ITO substrates retain their original fine-crystalline texture on the edge. Overall, plain ITO roughness and grain size vary slightly for different suppliers (Figure S5), the electrical and optical properties of ITO layers are comparable and efficiently meet the requirements for perovskite solar cell applications (Table S1). Assuming a thermal diffusivity of 0.018 cm^2^ s^–1^ and considering the pulse duration of the applied pulses was 200 ns (which is standard for industrial applications), the thermal diffusion length in the ITO can be approximated to ∼2 μm.^53^ This strongly supports the claim that photothermal ablation is the process that dictates the laser-matter interaction, characterized by heat-affected zones and the creation of macroscopic textures. Surface smoothing, grain coarsening, and melting zones with micrometer-sized grains replacing the characteristic nanostructure of pristine ITO were obtained. Cross-sectional views reveal the presence of melted regions at the edge of the ITO stripe with a thickness of up to 350 nm, while the standard thickness of the ITO layer is 180 nm, and an inclination angle of up to 26°. Moreover, the nonconductive part was found to be covered with a discontinuous ∼10 nm thick ITO layer. In contrast, CT-ITO revealed a gradually decreasing thickness starting from 120 nm toward the etched areas, forming a smooth, inclined termination zone (∼2.0–2.5 μm wide, ∼3.5–3.9° slope) without recrystallization or residual ITO on the etched regions.

**Figure 3:**
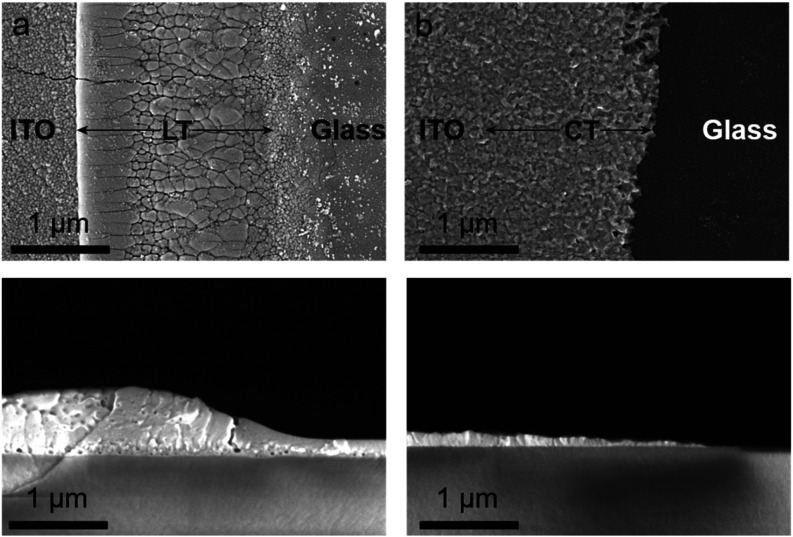
SEM images of ITO edge (top) and cross-section (bottom) for (a) LT-ITO and (b) CT-ITO.

SEM images and EDX elemental mapping of the ITO edges are shown in Figure 4a,b. A uniform indium distribution in the conductive layer with a steady superimposed silicon signal can be observed penetrating from underneath the ITO. As we approach the melted edge of LT-ITO (Figure 4a), the silicon signal intensity decreases, indicating a notable increase in the ITO thickness. Mapping the nonconductive layer shows that the visible features originate from residual ITO on the glass. EBSD mapping provides additional information on the evolution of microstructures during laser treatment. Several distinct regions can be distinguished when moving from pristine ITO to glass. The nontreated area has a pronounced preferred orientation and a high degree of intragranular misorientation associated with crystallographic stress. Then, closer to the melted layer, a thin band can be seen in which the local misorientation is significantly reduced due to high-temperature annealing and stress relief. Afterward, the melted region and ITO recrystallization begin, characterized by enlarged grains, the absence of crystallographic stress, and random grain orientations. Finally, a steep ITO termination is observed, followed by a ∼150 nm wide band of grains which end in complete amorphization, which is confirmed by cross-sectional views.

**Figure 4:**
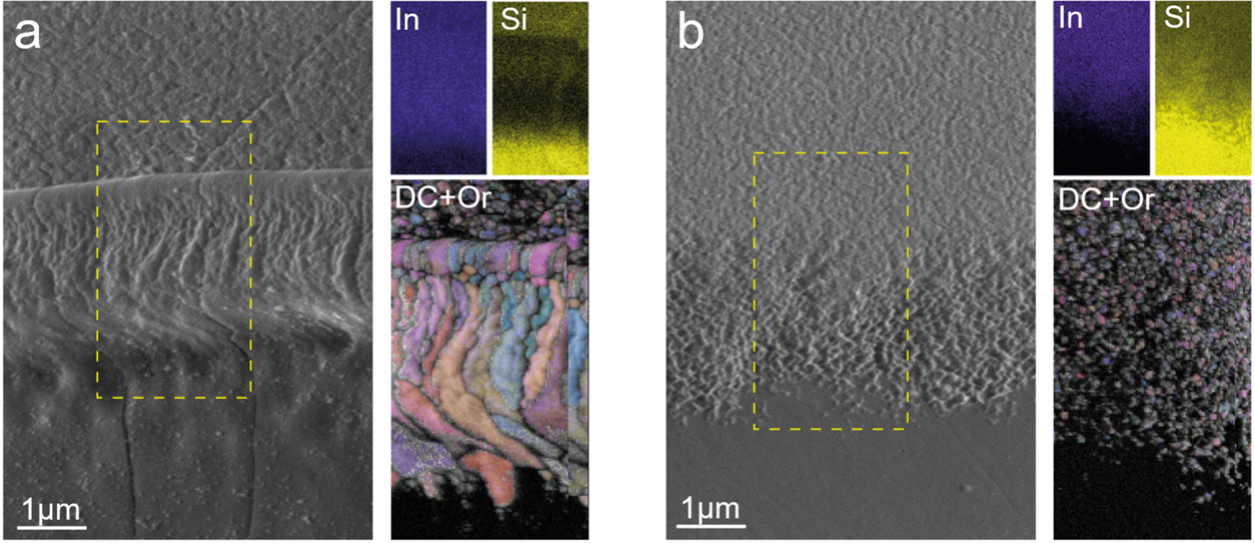
Top SEM image of ITO-glass edge, EDX map for indium and silicon, and Euler-colored grain orientation map superimposed on diffraction contrast for (a) LT-ITO and (b) CT-ITO. The dashed rectangle indicates the area for EDX/EBSD acquisition.

In contrast to these observations, CT-ITO substrates demonstrate no peculiarities: the size of ITO grains in the ITO stripe and transition layer is the same, with only negligible orientation randomization in the etched area. The diffracting area coincides with the visible ITO layer in the SEM images, and bare glass shows a characteristic picture of an amorphous material.

Since the local texture of the ITO in the termination region is different, it is most likely responsible for the formation of the degradation hotspots. We propose that during the early annealing stages of samples on LT-ITO, δ-CsPbI_3_ crystals are formed on the edges of the stripe. These needle-shaped δ-CsPbI_3_ crystals continue to grow, triggering the degradation of adjacent β-CsPbI_3_ perovskite grains via the mechanical pressure of the propagating δ-CsPbI_3_ crystals. This mechanical pressure arises from the differences in the crystalline structure of the two phases. Specifically, during the growth of the δ-CsPbI_3_ phase, the unit cell decreases in the *a* and *b* directions, but at the same time, there is a noticeable elongation of the *c* parameter. This induces mechanical pressure on the metastable β-CsPbI_3_ grains, overcoming the activation barrier of β- to δ-CsPbI_3_ transition, which is reported to be just ∼30 meV/atom and even lower, when the perovskite grains are subjected to stress.^54^ The propagation of the δ-phase in the [100] and [010] directions is slower than in [001]. Therefore, the hotspots present a radial-radiant structure and a visible semitransformed rim at the growth front. More resilient perovskite compositions, such as FAPbI_3_ and triple cation perovskites, exhibit a higher activation barrier (e.g., ∼52 meV/atom in the case of FAPbI_3_) and thus are substantially less sensitive to mechanical degradation triggers.^55^

To investigate the origin of the initial δ-CsPbI_3_ formation on the edges of LT-ITO, a CT-ITO substrate was treated with a laser beam while its fluence was gradually increased (laboratory laser-treated ITO, LLT-ITO). This enabled the creation of regions where the ITO grains melted (yet no ablation occurred due to the lower fluence) versus areas where the fluence level increased to ablate the ITO, which is always accompanied by a geometric alteration that locally increases the thickness of the ITO. A confocal height map reveals that high-fluence laser treatment effectively removed ITO from the glass surface, leaving uniform trenches with a depth of ∼180 nm, and elevated border lines with a side inclination of 100 nm μm^–1^ (Figure 5a). Optical microscopy of CsPbI_3_ film in polarized light shows a high correlation of bright δ-CsPbI_3_ with laser-treated areas (Figure 5b). Furthermore, the reconstructed height profiles were superimposed on the optical image, which allows the assignment of brighter δ-CsPbI_3_ zones and homogeneous dark brown β-CsPbI_3_ zones to different roughness areas and their correlation to the morphological features of ITO (Figure 5c). This comparison reveals a correlation between the first derivative of height and degradation (Figure 5d). Analysis of line profiles shows the emergence of degradation hotspots primarily at the line borders with a threshold inclination of at least 50 nm μm^–1^. Interestingly, no degradation occurs within the laser trenches, even though a rough, thin layer of molten ITO remains buried there, and the degradation hotspots that arise abruptly stop at the point where the laser-induced trench crosses the CT glass/ITO border, with no expansion to the sides (Figure S6). The same phenomenon is observed in molten planar ITO regions. On the other hand, a close inspection via EBSD of a rare gap between δ-CsPbI_3_-covered areas reveals only marginal differences in the average grain size or orientation of β-CsPbI_3_ on ITO, LT, or glass (Figure S7a,b). The only discriminative parameter is a slightly higher local misorientation, indicating the presence of internal crystallographic stress (Figure S7c). These observations suggest that the topography of the ITO layer at the LT termination region triggers the formation of degradation hotspots. The sharp decline in the layer thickness, on a scale comparable to the perovskite crystallite, may trigger the phase transition due to strain at the edge of the ITO layer or due to inhomogeneous heat transfer and colliding heat fluxes on the ITO edges. At the same time, a shallow decline in thickness, as is observed for CT-ITO, does not trigger this effect (Figure 5e,f). Consequently, while thicker interlayers, such as metal oxides or polymer films, can smoothen the sharp edges of LT-ITO, molecular layers cannot, triggering the rapid degradation in the case of SAMs being used. In contrast to CsPbI_3_, triple-cation perovskites (typically containing FA^+^, MA^+^, and Cs^+^) exhibit a higher structural tolerance to local strain and interfacial perturbations owing to their more favorable Goldschmidt tolerance factor and enhanced lattice flexibility. The partial substitution of smaller and larger A-site cations stabilizes the perovskite, suppressing the nucleation of nonperovskite phases. Consequently, triple-cation compositions display negligible performance differences on chemically and laser-patterned transparent electrodes, as their crystallization dynamics and defect tolerance mitigate the influence of local topographical or microstructural inhomogeneities that critically affect single-cation CsPbI_3_ films. This insight provides a new mechanistic understanding and a set of geometric constraints for future substrate design in inorganic CsPbI_3_ perovskite devices.

**Figure 5:**
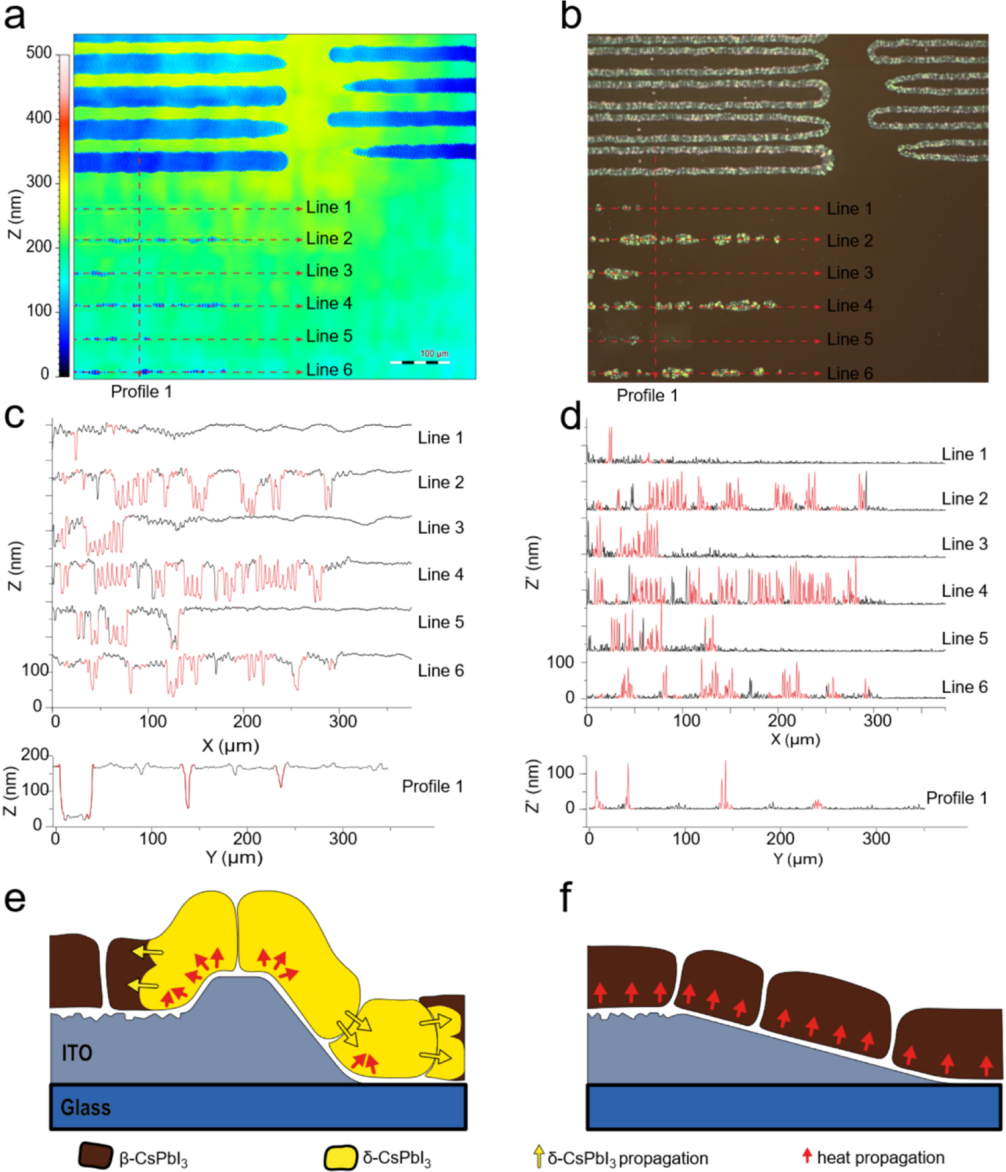
LLT-ITO substrate surface analysis. (a) Confocal elevation map, (b) CsPbI_3_ film in polarized light (bright spots correspond to δ-CsPbI_3_), and (c) height profiles reconstructed from corresponding lines on the elevation map, red segments correspond to δ-CsPbI_3_ phase, (d) modulus of the first derivative of the height profile, nm of inclination per μm in lateral direction, (e) model of δ-CsPbI_3_ emergence on the steep inclination due to uneven heating of the film and colliding heat fluxes inside β-CsPbI_3_ grains and (f) model of film formation on shallow slope (CT).

## Conclusions

This study reveals that the degradation of CsPbI_3_ into the δ-phase during layer formation is strongly influenced by the microstructural and topographical features of the underlying ITO substrate, particularly at termination regions formed by laser or chemical treatment. While chemically treated ITO enables the formation of high-quality layers, laser-etched ITO reproducibly triggers δ-CsPbI_3_ hotspot formation during annealing. Advanced characterization techniques, including SEM, EBSD, and confocal microscopy, demonstrate that localized recrystallization, grain coarsening, and nanoscale surface steps as small as 50 nm can initiate degradation. In contrast, neither sub-10 nm nor deep steps with minimal slope result in phase transition hotspot formation. Although the direct mechanism remains unclear, we propose that uneven heat transfer during the annealing step creates intragranular strain, and spots with high strain tend to undergo phase transitions at lower temperatures. These findings underscore the crucial role of substrate morphology and thermal history in determining perovskite stability. Overall, the work establishes a mechanistic basis for understanding how substrate morphology governs the intrinsic instability of inorganic perovskite films.

## Materials and Methods

### Materials

Chemically treated ITO-coated glass substrates (CT-ITO) were purchased from PsiOTech Ltd., while laser-treated ITO thin films (LT-ITO) were acquired from Liaoning Youxuan New Energy Technology Co., Ltd., China. Methylammonium iodide (MAI) and formamidinium iodide (FAI) were purchased from GreatCell Solar. Cesium iodide (CsI), 2-(3,6-dimethoxy-9*H*-carbazol-9-yl)­ethyl]­phosphonic acid (MeO-2PACz), bathocuproine (BCP), lead iodide (PbI_2_), and lead bromide (PbBr_2_) were purchased from TCI. DMAPbI_3_ was purchased from Xi’an Yuri Solar Co., Ltd. [6,6]-Phenyl C61 butyric acid methyl ester (PCBM) was purchased from Luminescence Technology Corp. Anhydrous dimethylformamide (DMF, 99.9%), and anhydrous dimethyl sulfoxide (DMSO, 99.8%) were purchased from Sigma-Aldrich.

### Solution Preparation

For triple cation solar cells, [Cs_0.05_(FA_0.87_MA_0.13_)_0.95_Pb­(I_0.9_Br_0.1_)_3_], we used a sequential solution method to prepare exactly stoichiometric 1 M precursor solutions. To do so, 2 M solutions of CsI, PbI_2_, and PbBr_2_ were prepared by dissolving each in a 4:1 volume ratio (v/v) mixture of DMF/DMSO and heating at 180 °C, and CsI in pure DMSO at 150 °C. Each solution was diluted by adding the appropriate solvent until the desired concentration of 1.155 M was reached, and then the CsI, PbI_2_, and PbBr_2_ solutions were mixed in a volume ratio of 0.05:0.85:0.15, yielding a 1.1 M solution of Cs_0.05_Pb­(I_1.75_Br_0.3_), which we term the inorganic stock solution. In two separate vials, FAI and MAI powders were added and weighed, into which the appropriate amount (0.95:1 molar ratio) of inorganic stock was added. This creates two new solutions, of the formula Cs_0.05_(FA_0.87_MA_0.13_)_0.95_Pb­(I_0.9_Br_0.1_)_3_. Finally, these two solutions were mixed in a 5:1 v/v ratio, in order to achieve the final molecular formula Cs_0.05_(FA_0.87_MA_0.13_)_0.95_Pb­(I_0.9_Br_0.1_)_3_.

For cesium-based solar cells, the CsPbI_3_ precursor solution was prepared by dissolving 0.8 M CsI and 0.8 M DMAPbI_3_ in DMF/DMSO (4:1) under active stirring for 12 h at room temperature.

### Device Fabrication

PV devices were fabricated in the device stack glass/ITO/MeO-2PACz/perovskite/PCBM/BCP/Ag. Depending on which perovskite and ITO were employed, a total of four systems were obtained: triple cation (Tr-cat-CT/LT) and cesium-based (CsPbI_3_–CT/LT). ITO substrates (CT or LT) were sequentially cleaned by sonication in 2% Hellmanex detergent, deionized water, acetone, and isopropyl alcohol. After blow-drying with pressurized nitrogen, the substrates were exposed to an oxygen plasma at 100 mW for 10 min to remove any residual contamination. Immediately after plasma cleaning, the substrates were transferred to a custom-built glovebox containing a dry-air atmosphere (<2% relative humidity), where MeO-2PACz was spin-coated from a 1.5 mg mL^–1^ solution in anhydrous isopropanol at 3000 rpm for 30 s, followed by a 10 min annealing step at 100 °C. After letting the substrates cool for 5 min, the perovskite layer was spin-coated: for triple-cation with a two-step recipe, first at 1000 rpm for 10 s, followed by 5000 rpm for 30 s. Then, 200 μL of anhydrous chlorobenzene were dripped onto the substrate 5 s before the end of the second step. The samples were annealed at 100 °C for 30 min and then transferred to a nitrogen-filled glovebox. For cesium-based, at 1000 rpm for 10 s and 4500 rpm for 30 s. Then the samples were first annealed at 30 °C for 2 min, then annealed at 180 °C for 15 min.

For the electron transport layer, PCBM was spin-coated dynamically from a 20 mg mL^–1^ solution in anhydrous chlorobenzene at 2000 rpm for 30 s, followed by a 10 min anneal at 100 °C. After letting the substrates cool for 5 min, BCP was spin-coated dynamically from a 0.5 mg mL^–1^ solution in anhydrous IPA at 4000 rpm for 30 s without annealing. To complete the devices, the samples were then transferred, without breaking the inert atmosphere, to a thermal evaporator where 80 nm silver electrodes were deposited at an initial rate of 0.01 nm s^–1^ for the first 10 nm, then 0.1 nm s^–1^ for the remaining. The whole active area for the devices was 0.045 cm^2^.

### Laser Processing

The laboratory laser-treated ITO (LLT-ITO) was obtained using a workstation equipped with a 60 W MOPA fiber laser (B4 model, ComMarker Technology Co., Ltd., China) with a maximum pulse energy of 2.0 mJ at a frequency of 30 kHz. The laser operated at a wavelength of λ_L_ = 1064 nm with a repetition rate of *f*_L_ = 20 kHz and a pulse duration of τ_L_ = 200 ns.

### Characterization Methods

A confocal microscope was employed to characterize the surface topography of the patterned ITO samples (Sensofar S Neox, Sensofar, Spain). The topographies’ surface profiles and average height values were calculated using SensoMAP Advanced Analysis Software (Sensofar, Spain). Atomic force microscopy (AFM) equipped with a Dimension ICON3 scanning probe microscope AXS S.S.S (Bruker Corporation) under ambient conditions in the ScanAsyst mode using a RTESPA-150 tip. Total transmittance was measured in the wavelength range from 400 to 850 nm using a spectrophotometer (Shimadzu UV-3600i Plus, Japan) equipped with an integrating sphere. The electrical characterization of the ITO substrates was conducted using the four-point probe method with a Keithley Sourcemeter (Sourcemeter 2450, Keithley).

X-ray diffraction (XRD) patterns were recorded using a SmartLab powder X-ray diffractometer (Rigaku, Japan), equipped with a HyPix 3000 2D X-ray detector in parallel beam setup in θ–2θ mode. A Cu kα rotating anode was used as X-ray source and all measurements were perfomed in air.

Scanning electron microscopy (SEM) images, energy-dispersive X-ray (EDX) maps and electron backscatter diffraction (EBSD) maps were collected with a Gemini 500 (ZEISS, Oberkochen, Germany) equipped with Oxford Ultim Max EDX spectrometer and Oxford NordLys Nano EBSD detector (Oxford Instruments, U.K.). Imaging was performed at 1.5 kV, EDX and EBSD data collection were performed simultaneously with an accelerating voltage of 12 kV as a compromise between data quality and electron penetration depth. EBSD maps were acquired using the Oxford AzTec software package and processed with Channel5 software package.

## Supplementary Material



## Notes

Additional figures (photographs of ITO substrates and perovskite films, XRD data, SEM and AFM images, optical microscopy images, and EBSD maps) and a table with the structural, optical, and electrical parameters of the employed ITO substrates (PDF)

## Abbreviations

ITO: indium tin oxide
LT-ITO: laser-treated ITO
CT-ITO: chemically treated ITO
LLT-ITO: laboratory laser-treated ITO
EBSD: electron backscatter diffraction
SEM: scanning electron microscopy
EDX: energy-dispersive X-ray spectroscopy
AFM: atomic force microscopy
IPA: isopropanol
BCP: bathocuproine
PCBM: [6,6]-phenyl C61 butyric acid methyl ester
PCE: power conversion efficiency
PSCs: perovskite solar cells.
