# Supplementary material for: Hidden Triggers of Degradation during Fabrication of Inorganic Perovskite Solar Cells
Source: ACS Appl Mater Interfaces. 2026 Feb 19;18(8):12661–9. doi: 10.1021/acsami.5c22948 (PMC12964337; doi:10.1021/acsami.5c22948)
Supplement: Supplementary file 1 [file am5c22948_si_001.pdf]

Supporting Information

Hidden Triggers of Degradation during Fabrication of Inorganic Perovskite Solar Cells

Vladimir Shilovskikh,[1,2] Herman Heffner,[1,2] Yitian Du,[1,2] Zongbao Zhang,[1,2] Fabian Paulus,[1,2] Boris Rivkin,[1,2] and Yana Vaynzof[1,2] *

[1]*Chair for Emerging Electronic Technologies, TUD Dresden University of Technology, Nöthnitzer Str. 61, 01187 Dresden, Germany*

[2]*Leibniz Institute for Solid State and Materials Research Dresden, Helmholtzstraße 20, 01069 Dresden, Germany.*

* Corresponding author. E-mail: y.vaynzof@ifw-dresden.de

Additional experiments including photographs of employed ITO substrates; photograph, XRD diffractogram, SEM and AFM figures of perovskite films; and a table with properties of employed ITO substrates

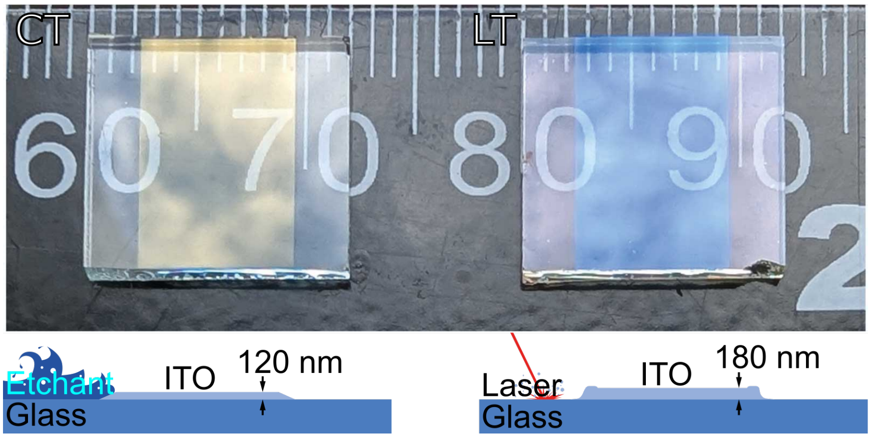

Figure S1. Photos of commercially available substrates, CT-ITO (left) and LT-ITO (right). Schematic cross-sections of the corresponding ITO substrates are shown on the bottom.

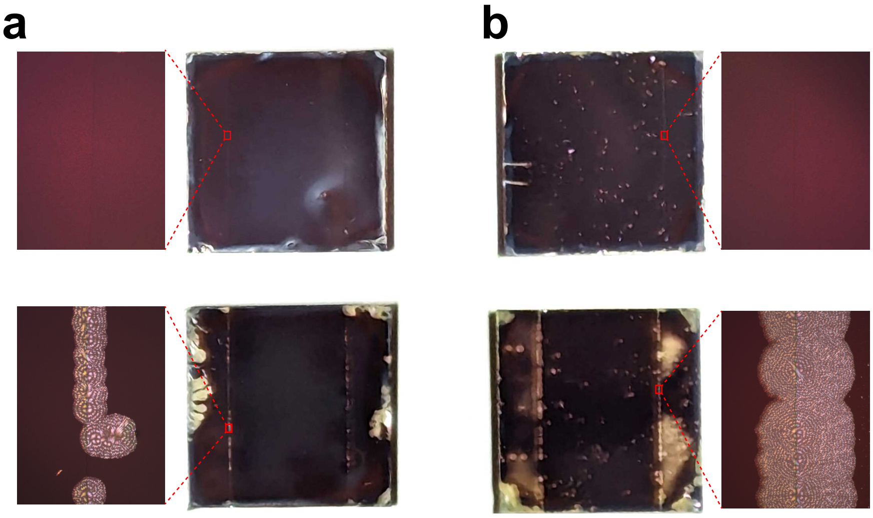

Figure S2. $CsPbI_3$ films, produced with modified recipes: (a) in N_2 atmosphere and (b) with a significant (30 minutes) delay between solution casting and annealing. Both films are annealed at 160 ^oC for 5 minutes.

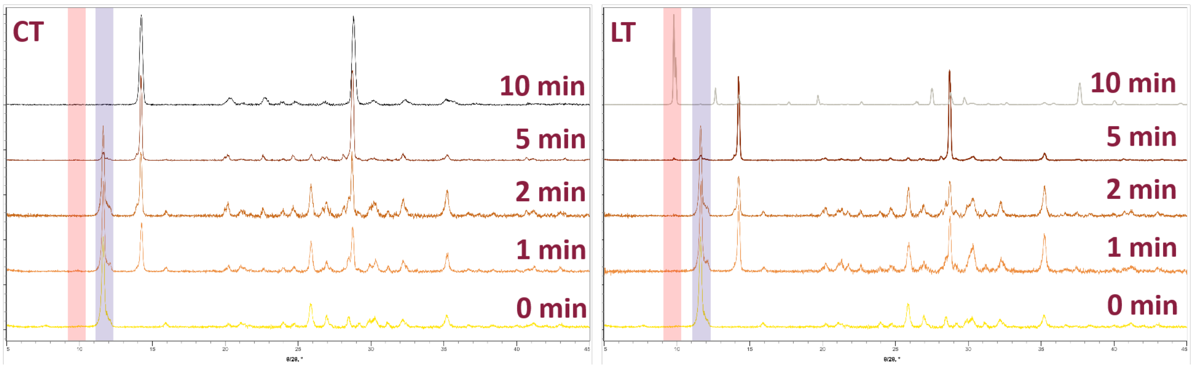

Figure S3. XRD patterns of $CsPbI_3$ films at different annealing durations for: CT-ITO (left) and LT-ITO (right). Red line highlights the position of the main δ-$CsPbI_3$ peak, while the dark line highlights the position of the intermediate phase.

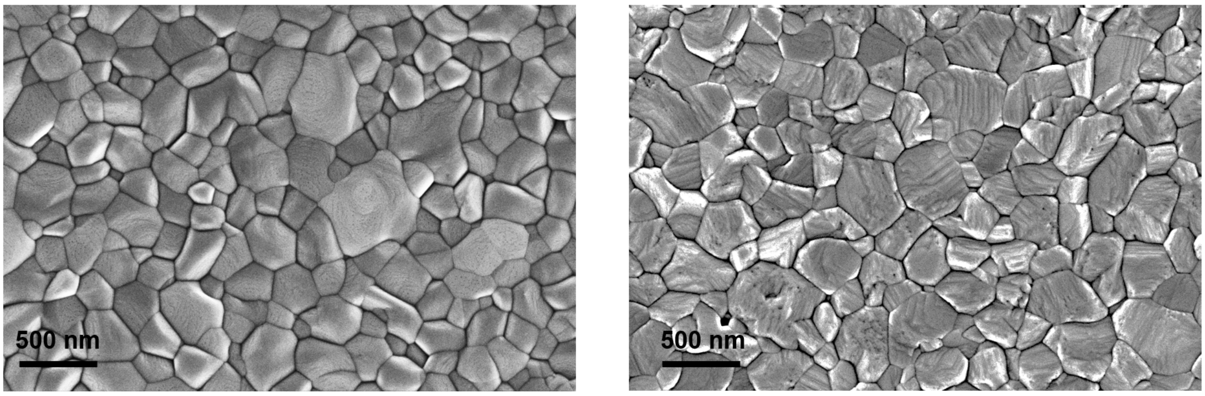

Figure S4. SEM images of β-$CsPbI_3$ on both LT (left) and CT (right) substrates. Images are acquired from the ITO-covered area, in the case of the LT sample, from the remaining dark region.

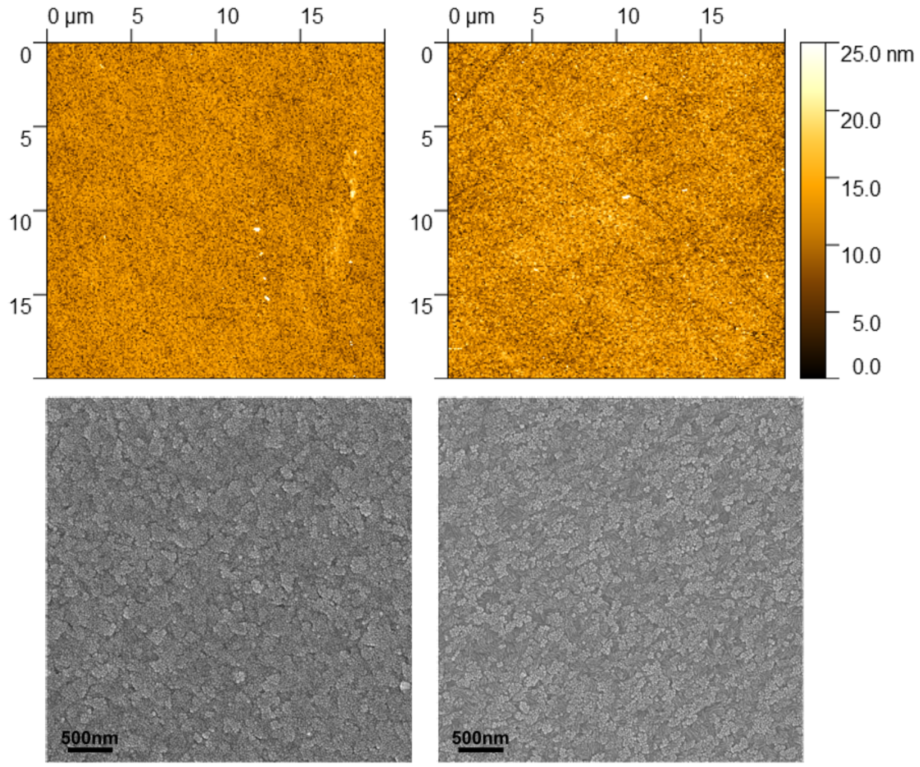

Figure S5.: Atomic force microscopy (AFM) images (top) and SEM images (bottom) for: CT-ITO (left) and LT-ITO (right).

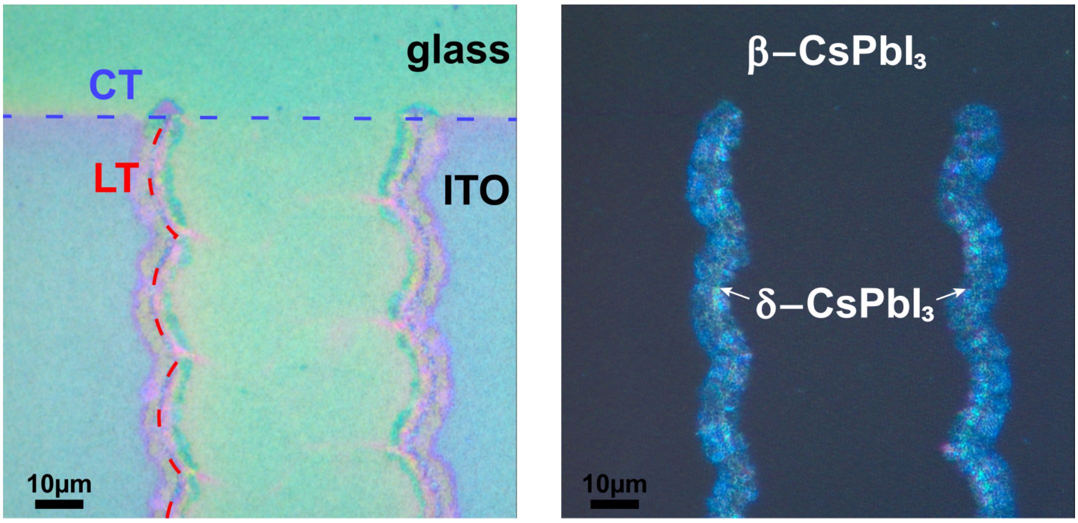

Figure S6. LT/CT intersection of LLT-ITO, covered with $CsPbI_3$ and annealed at 160 ^oC for 5 min. Brightfield microphotograph on the left and corresponding image in polarized light on the right.

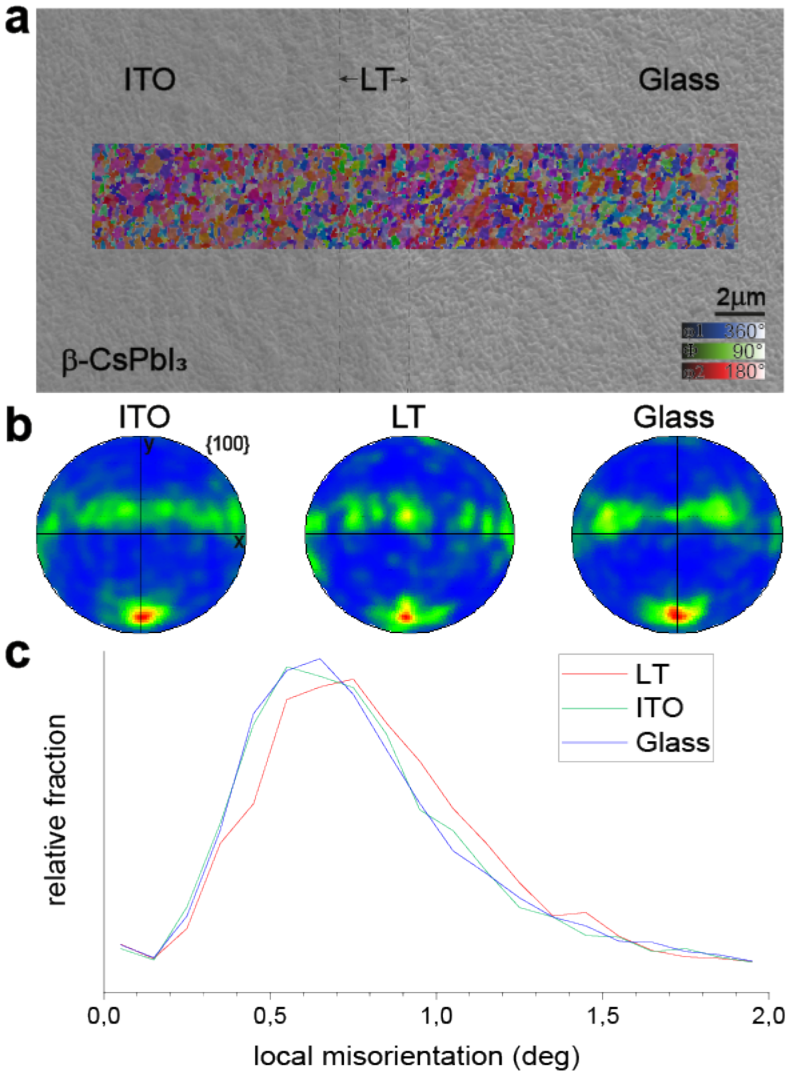

Figure S7. (a) EBSD map of non-degraded perovskite film across the LT termination; (b) orientation pole figures for the most representative {100} crystallographic orientation in cubic perovskite corresponding to different sections of EBSD map; (c) average local misorientation in perovskite grains measured as the highest orientation difference within a single grain on the scale of 3x3 pixels corresponding to different sections of EBSD map.

Table S1. Structural, optical, and electrical parameters of CT- and LT-ITO.

**Parameters**	**CT-ITO**	**LT-ITO**
Thickness [nm]	120	180
Roughness RMS [nm]	2.2	2.7
Average transmittance (400-850 nm)	85.1%	83.5%
Sheet resistance [ohm sq^{-1}]	11.4	8.5
